# The complete chloroplast genome sequence of *Hydrangea luteovenosa* (Hydrangeaceae)

**DOI:** 10.1080/23802359.2017.1347903

**Published:** 2017-07-11

**Authors:** Keum Seon Jeong, Jung Sung Kim, Hyuk-Jin Kim, Seung Hwan Oh, Kyung Choi

**Affiliations:** a Division of Forest Biodiversity and Herbarium, Korea National Arboretum, Pocheon, Korea;; b Research Institute for Dokdo and Ulleungdo Island, Kyungpook National University, Daegu, Korea

**Keywords:** *Hydrangea luteovenosa*, complete chloroplast genome, Hydrangeaceae, phylogenetic analysis

## Abstract

*Hydrangea luteovenosa* is a member of the family Hydrangeaceae. The complete chloroplast genome sequence of *H. luteovenosa* was characterized from MiSeq (Illumina Co.) pair-end sequencing data. The chloroplast genome of *H. luteovenosa* was 157,494 bp in length with a pair of inverted repeats (IRs) (25,126 bp) which were separated by a large single-copy (LSC) (86,596 bp) and a small single-copy regions (SSC) (18,646 bp). It contained 129 genes including 85 protein-coding genes, 36 tRNA genes, and 8 ribosomal RNA genes. The maximum-likelihood phylogenetic analysis with the previously reported chloroplast genomes showed that *H. luteovenosa* is most closely related to the tribe *Hydrangeeae*.

The genus *Hydrangea* (Hydrangeaceae) consists of 23 species that are mainly distributed in eastern Asia and northern America (McClintock 1957). It has been cultivated for gardening plants as well as pot-cultured and cut-flowers. *Hydrangea luteovenosa* usually grows in sunny areas such as the shrub forest around the forest edge of valley regions in Jeju-do Island province of South Korea and Japan. Chloroplasts play a key role in photosynthesis of green plants (Neuhaus and Emes 2000). The typical plant chloroplast genome consists of two large inverted repeats separated by a large single-copy (LSC) region and a small single-copy (SSC) region (Palmer 1985). Using the sequence data of chloroplast genomes, it could be applied to build up a super barcode for plants or to supply the genetic resources for the research of phylogeograhy, genetic diversity and evolution.

The study for *H. luteovenosa* has been focused on phylogenetic relationship with other species and there was no record of complete chloroplast genome sequence to date. In this study, we characterized a complete chloroplast genome sequence of *H. luteovenosa* and confirmed the phylogenetic relationship in the genus. The plant material was collected from Seogwipo-si (Jeju-do, South Korea).

The voucher specimen was deposited at the herbarium of Korea National Arboretum (KH). Total genomic DNA was extracted from ∼100 mg of fresh leaves using a DNeasy Plant Mini Kit (Qiagen Inc., Valencia, CA). After generating the pair-ended reads using the MiSeq platform (Illumina Co.) at Macrogen (http://www.macrogen.com/kor/), we assembled the chloroplast genome using Geneious R10.0.4 (Biomatters Ltd., Auckland, New Zealand). A map of the chloroplast genome was illustrated using OGDRAW (Lohse et al. 2013). The complete chloroplast genome of *H. luteovenosa* (MF370556) was 157,494 bp in length and contains a pair of inverted repeat (IR) regions of 25,126 bp, large single copy (LSC) region and small single copy (SSC) region with the lengths of 86,596 bp and 18,646 bp, respectively. It contained 129 functional genes including 85 protein-coding genes, 36 tRNA genes, and 8 rRNA genes. There were 16 protein-coding genes, 12 tRNA, and 8 rRNA genes, which were duplicated in the IR regions. The LSC region contained 61 protein-coding and 21 tRNA genes, whereas 11 protein-coding and 1 tRNA genes were included in the SSC region. Fourteen genes contained one or two introns including the protein-coding genes, *atpF, clpP, ndhA, ndhB, petB, petD, rpl2, rpoC1, rps12, rps16* and *ycf3*. The base composition is asymmetric (31.2% A, 18.4% C, 17.6% G, 32.7% T) with an overall GC of 36%. Phylogenetic analysis was performed using chloroplast coding gene sequences of *H. luteovenosa* and those of 14 related species of Hydrangeaceae family including *Loasa tricolor* (De Smet et al. 2015) as an outgroup. Maximum likelihood tree was generated using MEGA 6.0 (Tamura et al. 2013), and *H. luteovenosa* belonged to the tribe *Hydrangeeae* as expected. Interestingly it showed the most close relationship with *H. angustipetala* (Figure 1).

**Figure 1.**
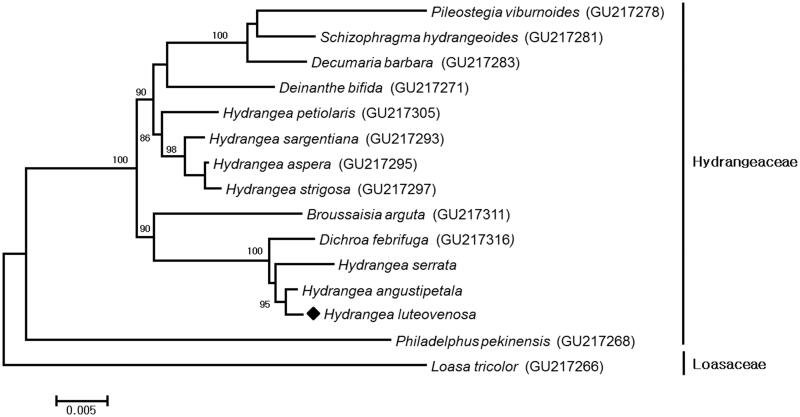
Maximum-likelihood phylogenetic tree of *H. luteovenosa* with 14 species belonging to the Hydrangeaceae based on chloroplast protein-coding sequences. Numbers in the nodes are the bootstrap values from 1000 replicates.
